# Supplementary material for: Conditional chemoconnectomics (cCCTomics) as a strategy for efficient and conditional targeting of chemical transmission
Source: eLife. 2024 Apr 30;12:RP91927. doi: 10.7554/eLife.91927 (PMC11060718; doi:10.7554/eLife.91927)
Supplement: Figure 3—source data 3. [file elife-91927-fig3-data3.zip › Figure 3 source data3 part 1.zip!/Figure 3-Source data 3-part1/1063f3-1-CRISPResso_Report_kboyr13q.zip!/CRISPResso_on_1063f3_4/2a.Nucleotide_percentage_quilt.pdf]

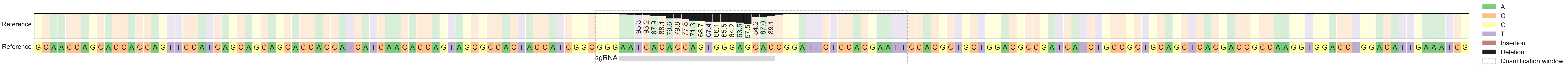
Reference
Reference
GCAACCAGCACCACCAGTTCCATCAGCAGCAGCACCACCATCATCAACACCAGTAGCGCCACTACCATCGGCGGGAATCACACCAGTGGGAGCACCGGATTCTCCACGAATTCCACGCTGCTGGACGCCGATCATCTGCCGCTGCAGCTCACGACCGCCAAGGTGGACCTGGACATTGAAATCG
93.3 93.2 87.9 88.1 79.6 79.8 77.8 71.3 68.7 67.4 66.1 65.5 64.2 63.5 57.5 84.2 87.0 89.1
sgRNA
A
C
G
T
Insertion
Deletion
Quantification window
